# Quality of Surgical Case Notes at Dow University Hospital according to modified ANKLe score

**DOI:** 10.12669/pjms.294.3813

**Published:** 2013

**Authors:** Masood Jawaid, Nighat Bakhtiar, Abdul Khalique, Zubia Masood

**Affiliations:** 1 Dr. Masood Jawaid, MCPS, MRCS, FCPS, Assistant Professor Surgery and Incharge e-Learning, Dow University Hospital and Dow International Medical College,; 2 Dr. Nighat Bakhtiar, MBBS, Postgraduate Trainee Surgery, Dow University Hospital,; 3 Dr. Abdul Khalique, FCPS, Assistant Professor Surgery, Dow University Hospital and Dow International Medical College,; 4 Dr. Zubia Masood, FCPS, Assistant Professor Surgery, Hamdard College of Medicine and Dentistry Karachi – Pakistan.

**Keywords:** Case notes, Quality, ANKLe score

## Abstract

***Objective:*** To find out quality of surgical case notes according to modified Adjusted Note keeping and Legibility (ANKLe) score in Dow University Hospital.

***Methods:*** For this audit, medical records of all the patients admitted in Dow University Hospital surgery department were reviewed from February 2012 to April 2012. The modified ANKLe score (total 24) is formed by the combination of, the content (out of 20) and legibility (out of 4) to give an overall score out of 24. A score of at least 20 (content score 17/20; legibility score 3/4) is considered as acceptable. It means that a surgical record is legible and the majority of the essential content is recorded.

***Results:*** A total of 236 records were evaluated. Overall mean ± standard deviation (SD) of ANKLe score was 18.4± 2.1 out of maximum score of 24. Content and legibility has overall mean scores of 14.4 out of 20 and 3.9 out of 4 respectively. Only two variables, patient’s name and consultant on call were documented in 100% of records while the least documented variable were social history 2 (0.2%). Legibility scoring system provides that 218notes out of total set of 236 notes (that is 92.4% of overall notes) have achieved a score of 4. The benchmark of 80% was achieved in 26.1% for total ANKLe score, 6.8% for contents and 99.1% for legibility.

***Conclusion:*** Overall, quality of records is not good but legibility part scores exceptionally high.

## INTRODUCTION

One of the primary skills required for good clinical practice is good medical record keeping. The development of a new dimension in the context of health sector has required medical record to be considered as a back bone in the overall process. Its importance is confined not only to the care for day to day patients, but has been extended to the fields of research, audit and legal purposes of medical.^1^ The accountability for maintaining an accurate, complete and legible records lies with all the members of the medical team.^2^ In surgery, especially it is considered very essential to maintain good documentation in the operation note.^3^ Once the notes are being filed, they will not be altered and in case if any change in the patient’s condition or the plan of management has to be recorded, a following entry will be made in the records.

The Royal College of Surgeons of England (RCS Eng) has published guidelines that help in highlighting the importance of record keeping in surgery.^4^ According to these guidelines, surgeons have the responsibility to ensure the legibility, and completeness of all the medical records and the accepted credentials of the patients have to be included. If the surgical notes are appropriately documented, the advantage will extend not only to the patient’s post-operative management, but will also be beneficial for the subsequent care of patients that may sometimes be long after the initial management. 

Scoring system has been formulated on the basis of the guidelines of the Royal College of Surgeon of England with the name of ANKLe score (Adjusted Note keeping and Legibility)^5^ and CRABEL Score (names on its proposed authors: Crawford JR, Beresford TP, Lafferty KL).^6^Both the scoring systems determine the quality of records while the legibility of notes is not taken into account in the first one (CRABEL score). Emphasizing on both content and legibility of the records, the ANKLe recording system, on the whole serves itself as a purposive and quantitative judgment tool for maintaining the quality of surgical records. Illegible writing of physician can be a cause of inaccurate records, inappropriate payments, impending lawful matters, loss of moment in time and wealth and also can lead to a disturbance for the hospital administration.^7 ^One institute reported that over 61% of its support staff spent more than ten minutes clarifying illegible orders.^8^

Accurate record keeping is one of the significant priorities of the Dow International Medical College Hospital affiliated with Dow University of Health Sciences. This audit is part of **a** big project of enhancing surgical record keeping practices of the hospital.^9^^,^^10^ At our institute duty doctors doesn’t have bleep with them. In order to make the scoring system relevant to local context one variable ‘doctor bleep’ is changed to ‘investigation documentation’ in the patients notes so that there will be no change is total score. Aim of this study was to assess the current status of documentation according to modified ANKLe score to identify the areas of improvement that will assure the enhancement of quality in this regard.

## METHODS

As a part of the record keeping audit, AK and NB reviewed the medical records with multiple international criteria^7^^,^^8^ of all the patients admitted in Dow University Hospital surgery department from February 2012 to April 2012. This study was about the quality of case note being assessed using the modified ANKLe Score.^5^

This scoring system was developed in 2008 with RCSE guidelines^4^ consisting of an initial part of 18 variables for initial clerking, each variable scoring 1 point. One variable was change from ‘doctor bleep’ to ‘investigation documented’ to align with the local context. In addition to this; two further points were included, specifically for the surgery department records. To determine the legibility a scoring system (1-4) was also included. The ANKLe score (total 24) is formed by the combination of, the content (out of 20) and legibility (out of 4) to give an overall score out of 24. A score of at least 20 (content score 17/20; legibility score 3/4) is considered as acceptable as done in the previous study.^6^ It means that a surgical record is legible and the majority of the essential content is recorded. 

Data was analyzed with the help of SPSS version 17 (SPSS Inc., Chicago, IL) for descriptive statistics.

## RESULTS

A total of 236 records were evaluated. Overall mean ± standard deviation (SD) of ANKLe score was 18.4± 2.1 out of maximum score of 24. Content and legibility has mean overall scores of 14.4 out of 20 and 3.9 out of 4 respectively (Table-I). 

Only two variables, patient’s name and consultant on call was documented in 100% of records while the least documented variable was social history 2 (0.2%), time when patient seen 8 (3.4%), referral source 14 (5.9%), and investigation documentations in records 20 (8.5%) Table-II. Legibility scoring system provides that 218 notes out of total set of 236 notes (that is 92.4% of overall notes) achieved a score of 4, indicating that quality of handwriting score is legible and clean (Table-II).

The benchmark of 80% was achieved in 26.1% for total ANKLe score, 6.8% for contents and 99.1% for legibility (Table-I)

**Table-I:** Modified ANKLe Score (n = 236).

*Standard*	*Mean ± SD of Score*	Standard Achieved* *(% of notes)*
Contents	14.4 ± 2.1	16 (6.8)
Legibility	3.9 ± 0.2	232 (99.1)
ANKLe	18.4 ± 2.1	61 (26.1)

* Standard of Content score 17/20; Legibility 3/4; Total ANKLe score 20/24

**Table-II:** Contents documentation in surgical case notes (n = 236).

*Generic content*		*Number (%) of documentation*
• Name		236 (100)
• Date of birth or hospital number		181 (76.7)
• Consultant on call		236 (100)
• Referral source		14 (5.9)
• Date seen		233 (98.7)
• Time seen		8 (3.4)
• Presenting complaint		213 (90.3)
• History of presenting complaint		206 (87.3)
• Past medical history		204 (86.4)
• Drug and allergy history		200 (84.7)
• Family history		202 (85.6)
• Social history		2 (0.8)
• Examination		191 (80.9)
• Working diagnosis		226 (95.8)
• Plan of care		120 (50.8)
• Investigations documented		20 (8.5)
• Doctor name		228 (96.6)
• Doctor signature		225 (95.3)
General Surgery specific content		
• Informed consent form		223 (94.5)
• Operative Notes form		218 (92.4)
Legibility scoring system		
* Quality of handwriting Score*		
Largely illegible	1	1 (0.4)
Legible with difficulty	2	1 (0.4)
Legible	3	16 (6.8)
Legible and neat	4	218 (92.4)

## DISCUSSION

Many comic stories are famous about the writing styles of doctors. However, our study supports the contrary view as is evident by the results of the study which showed that the legibility of most of the hospital records were excellent. Overall notes are not quite good in respect to total ANKLe score (only 1/4^th^ reach benchmark 80% total score). Most of studies about medical records only take into account the contents of the records not their quality in term of legibility. Poor legibility of physicians writing seriously effects the patient care, either by badly written prescriptions or by improper signatures, and can be due to inaccurate daily notes or incompletely written recommendation letters. Each and every person related directly to doctor’s notes like Pharmacists, nurses and other physicians must be able to read and understand it effectively. A large study of record keeping system in government teaching hospitals of Karachi reported that majority of records were deficient in temperature, pulse rate, blood pressure, diagnosis, weight, prescribed dose, history of patient, laboratory findings, previous record and discharge report.^11^ Another study^12^ reported same kind of findings.

It is well known in the world of medical records that if it is not written, it did not happen. But what if it is unreadable? In a study published in BMJ, a comparison of handwriting between doctors and other healthcare workers and administrators was done; in which the handwriting of doctors was found to be worst.^13^ A comparative study of doctors and community volunteers, doctors wrote more malformed individual alphabets.^14 ^Poor legibility by doctors is much more dangerous than by other qualified people.^13^ The unreadable job application or a marketing report is very different from an unreadable unconscious patient’s past medical history. However some studies are there which did not support this point of view. A study compared the legibility of doctors' handwriting with that of several other non‐healthcare occupations after adjustment for age, educational status and gender. There was no difference between doctors' handwriting and other professions.^15^ By means of poor handwriting someone can hamper the valuable statements and can even put in danger the life of any patient.^16^^,^^17^ However, our results are very good at least in terms of legibility. One of the main reasons may be that these records **were** all inpatient records which are mostly maintained by junior doctors whose writing is much legible as compared to consultants. We think, if our study had included records of outpatient department as well, which mostly written by consultant themselves; our results will be much different in term of legibility.

One of the least documented area in our study is social history part (0.2%), the reason being this heading is absent in history proforma currently under hospital use. This emphasizes the utilization of good quality of printed proformas.^18^Same is true for ‘referral source’ as accommodation Form doesn’t have space for this. It is usual practice that date was written on medical records but hardly any doctor has the habit of writing time in the progress notes (Table-II). All these important information can be easily improved with simple awareness of doctors and highlighting their importance.

Patient records are mostly documented under the time pressure which ultimately leads to poor legibility.^14^ Writing with a fountain pen is recommended to improve legibility ^18^ and the use of rubber stamp,^19^ with the name, position and pager number of the doctor noticeably. The notes of doctors become more legible after an easy and straightforward counseling to those who had bad writings.^5^ Being in the modern era the electronic medical records has taken place of hand-written notes but it’s not possible to avoid the handwritten documentation totally.

The success of Printed Performa to aid clinical communication has already been shown and proven by many studies.^20^^-^^22^ Through simplifications with check boxes the printed Performa decreases the quantity of handwriting, which ultimately decreases the misunderstanding of information due to poor handwriting. There is also evidence that simple educational intervention did improve the legibility of signature.^7^

One limitation of our study is that they its findings cannot be generalized as it is a single center study with small sample size. The low total modified ANKLe score is an area of concern for us and we will be using small educational activities in this regards to train our doctors. There is need for more studies about medical records by different institutes in view of improvement and where ever possible electronic medical records system should be used to improve patient care.

## CONCLUSION

The use of ANKLe score in assessing medical records has been demonstrated in this study. Both the complete content and legibility are very important part for high-quality medical record keeping. Overall, quality of records is not good but legibility part scores exceptionally high. The ANKLe score is one of the best assessing tools to find out the quality of notes because it includes both the content and legibility of clinical notes.

## Notes

***Source of Funding:*** None.

## References

[1] Chamisa I, Zulu BM (2007). Setting the records straight--a prospective audit of the quality of case notes in a surgical department. S Afr J Surg.

[2] Jawaid M, Qureshi MA, Arshad M, Masood Z (2010). Daily progress notes by surgical interns: An assessment of quality. Pak J Med Sci.

[3] Jawaid M, Masood Z, Humayun S (2011). Operative notes: a simple yet effective teaching resource for training. Pak Armed Forces Med J.

[4] The Royal College of Surgeons of England Good Surgical Practice.

[5] Dexter S, Hayashi D, Tysome JR (2008). The ANKLe score: an audit of otolaryngology emergency clinic record keeping. Ann R Coll Surg Engl.

[6] Crawford JR, Beresford TP, Lafferty KL (2001). The CRABEL score – a method for auditing medical records. Ann R Coll Surg Engl.

[7] Glisson JK, Morton ME, Bond AH, Griswold M (2011). Does an education intervention improve physician signature legibility? Pilot study of a prospective chart review. Perspect Health Inf Manag.

[8] Boehringer PA, Rylander J, Dizon DT, Peterson MW (2007). Improving the quality of the order-writing process for inpatient orders in a teaching hospital. Quality Management in Healthcare..

[9] Jawaid M, Khalique A, Moosa FA, Bakhtiar N (2012). Surgical Case Notes Quality by CRABEL Score in Dow University Hospital. J Postgrad Med Inst.

[10] Bakhtiar N, Khalique A, Moosa FA, Jawaid M (2012). Are we achieving the bench mark of surgical clinical case notes at Dow University Hospital?. Pak J Surg.

[11] Aziz S, Rao MH (2001). Existing record keeping system in government teaching hospitals of Karachi. J Pak Med Assoc.

[12] Mahmood K, Shakeel S, Saeedi I, Din Z (2007). Audit of medical record documentation of patients admitted to a medical unit in a teaching hospital NWFP Pakistan. J Postgrad Med Inst.

[13] Lyons R, Payne C, McCabe M, Fielder C (1998). Legibility of doctors’ handwriting: Quantitative comparative study. BMJ.

[14] Berwick DM, Winickoff DE (1996). The truth about doctors' handwriting: a prospective study. BMJ.

[15] Schneider K, Murray C, Shadduck R, Meyers D (2006). Legibility of doctors’ handwriting is as good (or bad) as everyone else’s. Qual Saf Health Care.

[16] Charatan F (1999). Family compensated for death after illegible prescription. BMJ.

[17] Bruner A, Kasdan M (2001). Handwriting errors: harmful, wasteful and preventable. J Ky Med Assoc.

[18] Hall R, Balachandren N, Aujla B, Haloob M, 2012 Improving admission records: use of clerking proforma for gynaecology patients. cited on 2013 May 10.

[19] Dunea G (1999). Beastly handwriting. BMJ.

[20] Medford ARL, France AJ (2004). Pocket-size self-inking rubber stamps improve legibility of case notes. Qual Primary Care.

[21] Osborn GD, Pike H, Smith M, Winter R, Vaughan-Williams E (2005). Quality of clinical case note entries: how good are we at achieving set standards?. Ann R Coll Surg Engl.

[22] Thompson AG, Jacob K, Fulton J, McGavin CR (2004). Do post-take ward round proformas improve communication and influence quality of patient care?. Postgrad Med J.

[23] Hobson JC, Khemani S, Singh A (2005). Prospective audit of quality of ENT emergency clinic notes before and after introduction of a computerized template. J Laryngol Otol.

